# Electroacupuncture Delays Hypertension Development through Enhancing NO/NOS Activity in Spontaneously Hypertensive Rats

**DOI:** 10.1093/ecam/nen064

**Published:** 2011-02-17

**Authors:** Hye Suk Hwang, Yoo Sung Kim, Yeon Hee Ryu, Ji Eun Lee, Young Seop Lee, Eun Jin Yang, Sun-Mi Choi, Myeong Soo Lee

**Affiliations:** Department of Medical Research, Korea Institute of Oriental Medicine, 461-24, Jeonmin-dong, Yuseong-gu, Daejeon 305-811, Republic of Korea

## Abstract

Using spontaneously hypertensive rats (SHR), this study investigated whether electroacupuncture (EA) could reduce early stage hypertension by examining nitric oxide (NO) levels in plasma and nitric oxide synthase (NOS) levels in the mesenteric resistance artery. EA was applied to the acupuncture point Governor Vessel 20 (GV20) or to a non-acupuncture point in the tail twice weekly for 3 weeks under anesthesia. In conscious SHR and normotensive Wistar Kyoto (WKY) rats, blood pressure was determined the day after EA treatment by the tail-cuff method. We measured plasma NO concentration, and evaluated endothelial NO syntheses (eNOS) and neuronal NOS (nNOS) protein expression in the mesenteric artery. Systolic blood pressure (SBP) and diastolic blood pressure (DBP) were lower after 3 weeks of GV20 treatment than EA at non-acupuncture point and no treatment control in SHR. nNOS expression by EA was significantly different between both WKY and no treatment SHR control, and EA at GV20 in SHR. eNOS expression was significantly high in EA at GV 20 compared with no treatment control. In conclusion, EA could attenuate the blood pressure elevation of SHR, along with enhancing NO/NOS activity in the mesenteric artery in SHR.

## 1. Introduction

Electroacupuncture (EA) inhibits sympathetic effects by regulation of nitric oxide synthase (NOS) expression in the central nervous system [1–3]. NO, which is produced by vascular endothelial cells from its precursor, is a potent vasodilator and plays an important anti-hypertensive role in blood pressure (BP) homeostasis. The depressor effect of EA on BP is primarily caused by vasodilation of the mesenteric vessels due to inhibition of sympathetic vasoconstrictor tone [4].

In spontaneously hypertensive rats (SHR) and other animals with genetic hypertension, morphological or functional changes within the arterial wall may result in an increased peripheral vascular resistance, thereby leading to hypertension [5, 6]. These processes in SHR bear a resemblance to that of essential hypertension in humans. Thus, SHR are widely used as a model to study the mechanism, pathophysiology and management of essential hypertension. SHR have irregularities in several vasoregulatory factors, including an impaired NO/NOS system. Therefore, SHR are a beneficial model to investigate the mechanism of action responsible for the effects of acupuncture in the treatment of essential hypertension.

Underlying mechanism of anti-hypertensive effect of EA by adjustments of total peripheral resistance which affect the vascular regulatory system and BP has not been well understood as much as that by regulation through the central nervous system [7]. The aim of this study was to investigate whether EA could reduce early stage hypertension by examining NO levels in plasma in SHR and NOS levels in the mesenteric resistance artery.

## 2. Methods

### 2.1. Animals

A total of 24 4-week-old male SHR/Izm and age-matched six Wistar Kyoto rats (WKY/Izm) were purchased from Japan Shizuoka Laboratory Center (Shizuoka, Japan) and used after acclimatization for 1 week. We used WKY rats as the normotensive control group because they are genetically homogeneous. The animals were given food and water *ad libitum*. They were housed at a controlled ambient temperature of 22–25°C with 55 ± 5% relative humidity and a 12 h light/12 h dark cycle (lights on at 8:00 AM). Rats were acclimatized into BP measuring conditions for at least 1 week before undergoing EA treatments. After measuring BP, SHR were randomly divided into three groups: untreated group (*n* = 6), EA at GV20 (*n* = 6) and EA at non-acupuncture points group (*n* = 6). Animal experiments were carried out in accordance with the National Institute of Health's Guide for the Care and Use of Laboratory Animals, and experimental procedures were approved by the Institutional Animal Care and Use Committee at the Korea Institute of Oriental Medicine.

### 2.2. Measurement of BP

Systolic arterial blood pressure (SBP) and diastolic blood pressure (DBP) were measured non-invasively by the tail-cuff method using the Visitech BP-2000 BP Analysis System (Visitech Systems, Apex, NC, USA) on the next day of EA treatment. To ensure accuracy and reproducibility, the rats were trained for 1 week prior to the experiment, and measurements were taken at the same time each day. Unanesthetized rats were placed on the specimen platform, and their tails were placed through tail cuffs and secured in place with tape. Following a 10 min warm-up period, 10 preliminary cycles were performed to allow the rats to adjust to the inflating cuff. For each rat of each group, four cycles were recorded at each time point without intervention from moving by environmental situations.

### 2.3. EA Stimulation

EA was applied to the acupuncture point GV20 or to a non-acupuncture point in the tail on the first and fourth day of the week for 3 weeks (EA applied from 6th to 8th week) with a pair of bipolar stimulation electrodes after placing the rats under isoflurane anesthesia for reducing stress of electrical stimulations (in the flow of mixture of oxygen and nitrous oxide; 3% for induction and 1.5% for maintenance). GV20 is located at the vertex, on the dorsal midline. The methods to determine acupoints in rat is the transpositional method, which locates the veterinary acupoints by transforming human acupoints onto animal anatomy [8]. According to this, the acupoints in tail is considered as the most suitable control points for avoiding possible effects of EA.

Two stainless steel acupuncture needles (0.15 mm diameter; DB needle CO, LTD, Korea) were mounted in a holder with 1 mm separation between the tips. The needle set was vertically inserted to a depth of 4 mm (cutaneous and muscle) at the GV20 point on the midsagittal line between the both of temporalis muscles of rat (equivalent to the epicranius muscle of human), at the intersection of a line connecting the right and left ear apices. As a control, a non-acupuncture point located at the junction between the tail and buttock was also applied with the same parameters. Electrical stimulation was performed using a Grass S88 stimulator (Grass Instrument Co., West Warwick, RI, USA) connected to the pair of needle electrodes. Electrical stimuli with 10 Hz frequencies, 1 mA and 1 ms duration pulses were applied to the acupuncture point. The current delivered was monitored at all times, and the polarity was reversed every 60 s to prevent polarization of the electrodes. The total duration of EA stimulation was 10 min. After the termination of EA, anesthesia was immediately discontinued, and the rats usually resumed full activity within 5 min.

### 2.4. Tissue Preparation

The animals were anesthetized with an overdose of sodium pentobarbital (50 mg/kg body weight) by intraperitoneal injection on the last day of EA treatment, and perfused intracardially with phosphate-buffered saline (PBS: pH 7.4) (in millimolar): 140 NaCl, 3 KCl, 10 Na_2_HPO_4_, 2 KH_2_PO_4_. Because change of vascular tone in the resistance-sized artery contribute to the peripheral resistance and regulate the BP, the mesenteric arteries of four randomly selected rats in each group were carefully dissected out, cleared of connective tissue and flushed out with modified Krebs-Henseleit solution (KHS) at 4°C; the KHS had the following composition (millimolar): 119 NaCl, 4.7 KCl, 2.5 CaCl_2_, 24 NaHCO_3_, 1.18 KH_2_PO_4_, 1.2 MgSO_4_, 0.01 EDTA, 5.5 glucose. Tissues were immediately snap-frozen in liquid nitrogen and stored at −70°C until processed.

### 2.5. Measurements of Total Nitrate and Nitrite

Blood samples were collected from cardiac puncture into a polypropylene tube containing sodium-EDTA (3–7 mg ml^−1^ of blood) as an anti-coagulant, just prior to tissue preparation on the last day of EA treatment. Samples were centrifuged for 15 min at 3000 g, and plasma was removed and stored at ≤−70°C until use. As blood hemoglobin and other protein components of plasma interfere with the spectrophotometric value at 540 nm, samples were centrifuged at 14 000 g at 4°C for 30 min with 10 kDa molecular weight cut-off filters (Millipore Micron YM-10, Bedford, USA). Generation of NO was determined by measuring nitrite accumulation in plasma samples using a commercially available Griess reagent containing 1% sulphanilamide and 0.1% *N*-(1-naphthyl)-ethylenediamine dihydrochloride (R&D systems, Minneapolis, MN, USA). Sample and Griess reagent (50 *μ*l of each) were mixed and incubated for 5 min, and absorption was determined on an automated microplate reader (Synergy HT, BIO-TEK) at 540 nm. Sodium nitrate and nitrite standards were used to generate a standard curve for quantification: the final concentrations ranged from 0 to 200 *μ*M.

### 2.6. Western Immunoblotting Analysis

The mesenteric arteries were ground in a mortar containing liquid nitrogen. The powdered tissue was suspended in 100 *μ*l of lysis buffer (20 mM Tris-HCl, 150 mM NaCl, 5 mM EDTA, 0.1% SDS, pH 7.5) containing protease inhibitors (10 *μ*g ml^−1^ of aprotinin, 1 mmol l^−1^ PMSF and 10 *μ*g ml^−1^ of leupeptin) and agitated at 4°C for 30 min. After centrifugation at 17 900 g at 4°C for 30 min, the protein concentration in the supernatant was determined using a Bradford protein assay kit (Bio-Rad Laboratories Inc., CA, USA). These measurements were performed to determine the eNOS and nNOS protein mass. Briefly, thoracic aorta and mesenteric artery tissue preparations (30 *μ*g of protein for nNOS and eNOS) were size fractionated on a 10% SDS-PAGE gel at 120 V for 2 h. After electrophoresis, proteins were transferred onto a nitrocellulose transfer membrane (Whatman GmbH) at 200 mA for 120 min using the Bio-Rad transfer system. Membranes were blocked with Tris-buffered saline buffer (TBS), pH 7.4, and 5% skimmed milk, then incubated overnight at 4°C with mouse monoclonal anti-nNOS, anti-eNOS (1 : 2500 dilution, BD Transduction Laboratories, CA, USA), or anti-*β*-actin (1 : 1000, AbCAM, Cambridge, UK) in TBS. *β*-Actin was used as an internal control. The membrane was then washed for 30 min in a shaking bath, with the wash buffer (TBS buffer containing 0.1% Tween-20) changed every 5 min. The membranes were subsequently incubated with a horseradish peroxidase-conjugated anti-mouse IgG antibody (1 : 1000, BD Transduction Laboratories) at room temperature. The washes were repeated before the membrane was developed with a light-emitting, non-radioactive method using ECL reagent (Amersham Inc., Buckinghamshire, UK). The membrane was then subjected to autoluminography for 1–5 min. The relative optical density of the respective bands was quantified by densitometric scanning of the blots using i-Solution DT image analysis software (Image and Microscope Technology).

## 3. Statistical Analysis

All data are expressed as mean ± SEM. Statistical significance was assessed using one way ANOVA followed by an all pair-wise multiple comparison procedure (Turkey's test). Differences were considered statistically significant at values of *P* < .05.

## 4. Results

### 4.1. Relationship between Age and BP

From ages 4 through 12 weeks, the SBP (mean ± SEM) increased progressively from 152.1 ± 3.02 mmHg to 206.8 ± 6.06 mmHg in SHR, but not in WKY (Table 1). Also, the DBP (mean ± SEM) increased progressively from 119.7 ± 5.52 mmHg to 174.3 ± 1.63 mmHg in SHR rats. Both SBP and DBP were significantly higher than those of age-matched WKY from ages 4 through 12 weeks.

### 4.2. EA Treatment on GV20 Significantly Delays the Development of Hypertension in SHR

**Figure 1:**
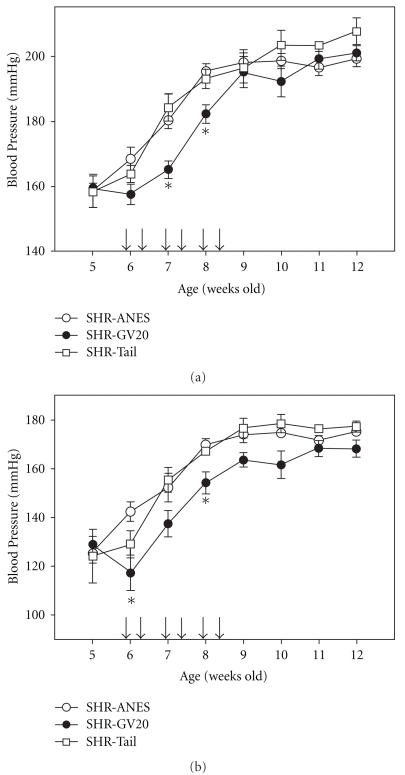
(a) SBP in SHR that were anesthetized for the same period of EA stimulation (SHR+ANES), in Governor Vessel 20 (GV20) treated rats (SHR-GV20), or in non-acupoint treated rats (SHR-Tail) from 5 to 12 weeks. (b) Diastolic blood pressure in SHR (SHR+ANES), GV20 (SHR-GV20) or tail acupuncture groups (SHR-Tail) from 5 to 12 weeks. Results are mean ± SEM for six rats in each group. *P < .05, compared with SHR+ANES. The arrows show the EA treatment for 3 weeks (6–8 weeks).

The SBP of 8-week-old SHR was higher than that of the age-matched WKY as shown in Table 1. And the SBP of 4-week and 12-week-old SHR were higher than those of the age matched WKY as listed in Table 1. EA significantly attenuated BP in SHR rats (Figure 1), but not to levels of age-matched untreated WKY as shown in Table 1. SBP at 8 weeks increased progressively to 195.38 ± 2.34 and 193.02 ± 2.97 mmHg in SHR and non-acupuncture point treated group, respectively, while GV20 treatment reduced SBP to 182.2 ± 2.87 mmHg in SHR at the same age. DBP at 8 weeks increased progressively to 169.79 ± 2.5 and 167.21 ± 1.26 mmHg in SHR and non-acupuncture point treated group, respectively, while GV20 treatment reduced DBP to 154.1 ± 4.61 mmHg in SHR at the same age (Figure 1(b)). EA significantly reduced BP in SHR rats (Figure 1), but not to levels of age-matched untreated WKY as listed in Table 1.

### 4.3. NOS Expression in WKY Rats and SHR after EA Treatments

**Figure 2:**
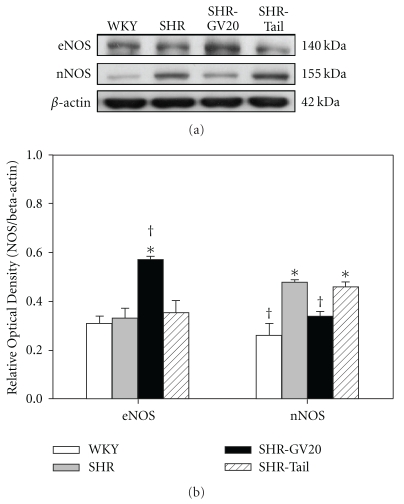
(a) Representative western blot of eNOS and nNOS protein in mesenteric artery from WKY and SHR, either control, EA-treated at GV20 (SHR-GV20) or non-acupoint (SHR-tail) conditions. (b) Lower panel shows densitometric analysis of the western blot of eNOS and nNOS protein. Relative abundance of NOS protein compared with *β*-actin. Results are expressed as mean ± SEM for four rats in each group. Results are expressed as mean ± SEM for four rats in each group. *P < .05, compared with WKY group; **^†^** *P* < .05, compared with no-treated SHR group.

We compared the expression of eNOS and nNOS in the mesenteric artery of SHR and WKY to delineate whether they play a differential role in the augmented level of NO by EA during hypertension. We determined the expression of eNOS and nNOS protein in the mesenteric arteries of 8-week-old rats by western blotting and measuring band intensities (Figure 2). The developmental stage of hypertension did not significantly affect the eNOS expression level. eNOS band intensities relative to the corresponding *β*-actin band were 0.31 ± 0.03 (WKY group), 0.33 ± 0.04 (SHR group), 0.57 ± 0.01 (SHR-GV20 group) and 0.35 ± 0.05 (SHR-Tail group). EA treatment increased the eNOS expression associated with delaying development of hypertension and showed even higher eNOS expression than the non-acupuncture point treated SHR and non-treated SHR groups in the mesenteric arteries. For nNOS, expression (relative to *β*-actin) was 0.26 ± 0.05 (WKY group), 0.48 ± 0.01 (SHR group), 0.34 ± 0.02 (SHR-GV20 group) and 0.46 ± 0.02 (SHR-Tail group). nNOS expression in SHR was significantly higher than age-matched WKY rats, and EA treatment significantly decreased it (*P* < .05) (Figure 2(b)).

### 4.4. Effect of EA on Plasma Nitrate/Nitrite Levels

**Figure 3:**
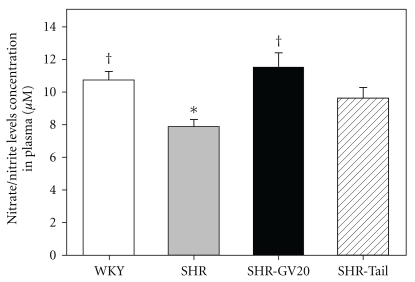
Plasma nitrate/nitrite levels in WKY and SHR under control- or EA-treated at GV20 (SHR-GV20) or non-acupoint (SHR-tail) conditions. Results are expressed as mean ± SEM for six rats in each group. *P < .05, compared with WKY group; **^†^** *P* < .05 compared with no-treated SHR group.

The basal plasma nitrate/nitrite level was significantly lower in SHR than WKY at 8 weeks (*P* < .05). EA treatment for 3 weeks significantly increased plasma nitrate/nitrite levels at 8 weeks of SHR (*P* < .05, Figure 3).

## 5. Discussion

The main findings of this study were that long-term treatment with EA delayed hypertension development, and this restored NO in the plasma of SHR. In this study, eNOS expression was also significantly increased by EA in mesenteric artery of SHR, whereas nNOS expression was significantly attenuated.

GV20 alone, or in combination with other acupuncture points, is used for the treatment of hypertension and pre-hypertension patients [9–11]. Scalp acupuncture decreases superoxide dismutase activity, reducing the oxidative stress reaction [12]. Kim et al. [7] reported that EA treatment on Tsu-san-li (ST36) controlled the NOS system in the stomach and cheek pouch tissues, which were on the stomach meridian, but did not control that in liver tissue, a non-stomach meridian organ in the two-kidney, one-clip renal hypertension hamster model. While examining the distribution of NO in the skin acupuncture points of rats, Chen et al. [13] showed that l-arginine-derived NO synthesis appears to mediate noradrenergic function on skin sympathetic nerve activation, which contributes to the skin electrical resistance of acupuncture points and meridians. The hypotensive action of NO induced by EA stimulation remains an unexplained but reproducible observation.

NO is a potent vasodilator that is necessary to maintain BP homeostasis. Similarly Briones et al. [14] also reported that nNOS expression is greater in mesenteric arteries of SHR than in those of WKY. In contrast, Forte et al. [15] reported that basal NO synthesis by endothelial cells is reduced in patients with untreated essential hypertension. Furthermore, Hatta et al. [16] reported that antihypertensive therapy increases the reduced basal NO levels in SHR and DOCA-salt rats. Mokuno et al. [17] have shown that NO production induced by mechanical stimulation was markedly reduced in 5-week-old SHR at the pre-hypertensive stage, and this impairment of nitric oxide production preceded the onset of hypertension in SHR. These reports support our findings that basal release of NO is reduced in SHR during hypertension.

The vascular generation of oxygen species was increased in development of hypertension [18] and eNO was rapidly inactivated in the presence of superoxide anions [19]. Neuronal NO metabolism by superoxide anions in mesenteric arteries from young SHR is also elevated [20]. Vaziri et al. [21] also reported that antioxidant therapy ameliorated hypertension and mitigated the upregulation of NOS in vascular and renal tissues. Furthermore, the increased oxidative stress and degradation of NO from eNOS has been described in SHR [22–24]. Together, these data suggest that anti-hypertensive treatment restores decreases in NO release by high BP and enhances NOS bioavailability in the aorta.

In hypertension, where intraluminal pressure, shear stress and oxidative stress are increased, augmented nNOS-derived NO may offset decreased eNOS-derived NO, thereby acting as an adaptive mechanism [25]. eNOS^−/−^ mice showed elevated BP in conscious states [26], confirming that NO derived from eNOS plays an important role in regulating BP as a vasodilator. But nNOS knockout mice, however, have enlarged stomachs and defects in the inhibitory junction potential involved in gastrointestinal motility, but not hypertension [27, 28]. In mice lacking functional eNOS^(−/−)^, acute injection of a non-selective NOS inhibitor, Nw-nitro-l-arginine (l-NNA), decreased mean BP [26], suggesting that NO derived from isoforms other than eNOS increases BP in the absence of eNOS activity [29]. These results support that the eNOS activation could affect to BPã without abnormally overactivated-nNOS activation inã SHR. Our result shows EA treatments for 3 weeks prefer enhancing eNOS activity to nNOS activity compensated eNOS deactivation.

The autonomic nervous system is involved in the development of hypertension, and chronic imbalance of the autonomic nervous system is a prevalent, potent risk factor for adverse cardiovascular events [30]. Both increased sympathetic nerve firing rates and reduced neuronal norepinephrine re-uptake contribute to sympathetic activation in hypertension [31]. Acupuncture stimulation seems to reduce sympathetic nervous system activation via activation of the cholinergic system or opioid receptors in the rostral ventrolateral medulla [4, 32]. NO in the central nervous system plays a very important role in the control of sympathetic outflow and regulation of cardiovascular activities, and EA stimulation can restore the NOS system in the central nervous system of stress-induced hypertension [33] and SHR [34] models. Microinjection of nNOS antisense oligos into the gracile nucleus produces a depressor and inhibitory cardiovascular response to EA stimulation [1]. Therefore, along with the cited findings, sympatho-inhibition and vasodilation may be induced by EA stimulation in various hypertension models.

It would be interesting to compare this result with clinical therapeutic effects of previous clinical trials. According to previous trials, acupuncture is more effective than sham only when given in addition to medication [11, 35] and acupuncture alone is not better than sham [36, 37]. These results can imply both acupuncture and sham acupuncture are effective for lowering BP. The result of our study shows that the suppressive effect of EA on BP seems to be an immediate one, without any cumulative effects. It would be useful to investigate the immediate effects of acupuncture for lowering BP in the future clinical settings.

In conclusion, our results support the concept that EA could attenuate the BP elevation of SHR, along with enhancing NO/NOS activity in the mesenteric artery in SHR. However, future studies are necessary to investigate the underlying NOS mechanisms of the peripheral autonomic nervous system in blood vessels.

## Funding

Acupuncture, Moxibustion, and Meridian Research Project (K08010) of the Korea Institute of Oriental Medicine.

## Figures and Tables

**Table 1:** The systolic and diastolic blood pressure in SHR and age-matched WKY rats at age 4, 8 and 12 weeks.

	SBP			DBP		
	4 weeks	8 weeks	12 weeks	4 weeks	8 weeks	12 weeks
WKY	130.8 ± 3.18	134.6 ± 1.95	130.9 ± 6.99	96.7 ± 5.34	101.9 ± 4.45	103.8 ± 5.62
SHR	152.1 ± 3.02*	193.1 ± 3.57*	206.8 ± 6.06*	119.7 ± 5.52*	163.9 ± 2.47*	174.3 ± 1.63*

Results are expressed as mean ± SEM for six rats in each group. *P < .05 compared with age-matched WKY.
